# Discrimination of lipid composition and cellular localization in human liver tissues by stimulated Raman scattering microscopy

**DOI:** 10.1117/1.JBO.29.1.016008

**Published:** 2024-01-24

**Authors:** Fiona Xi Xu, George N. Ioannou, Sum P. Lee, Christopher Savard, Christian L. Horn, Dan Fu

**Affiliations:** a University of Washington, Department of Chemistry, Seattle, Washington, United States; b Veterans Affairs Puget Sound Health Care System, Department of Medicine, Division of Gastroenterology, Seattle, Washington, United States; c University of Washington, Department of Medicine, Division of Gastroenterology, Seattle, Washington, United States; d Veterans Affairs Puget Sound Health Care System, Research and Development, Seattle, Washington, United States; e San Antonio Military Medical Center, Department of Medicine, Division of Gastroenterology and Hepatology, Fort Sam Houston, Texas, United States

**Keywords:** stimulated Raman scattering microscopy, human liver tissue, nonalcoholic fatty liver disease, fibrosing steatohepatitis, lipid

## Abstract

**Significance:**

The molecular mechanisms driving the progression from nonalcoholic fatty liver (NAFL) to fibrosing steatohepatitis (NASH) are insufficiently understood. Techniques enabling the characterization of different lipid species with both chemical and spatial information can provide valuable insights into their contributions to the disease progression.

**Aim:**

We extend the utility of stimulated Raman scattering (SRS) microscopy to characterize and quantify lipid species in liver tissue sections from patients with NAFL and NASH.

**Approach:**

We applied a dual-band hyperspectral SRS microscopy system for imaging tissue sections in both the C–H stretching and fingerprint regions. The same sections were imaged with polarization microscopy for detecting birefringent liquid crystals in the tissues.

**Results:**

Our imaging and analysis pipeline provides accurate classification and quantification of free cholesterol, saturated cholesteryl esters (CEs), unsaturated CE, and triglycerides in liver tissue sections. The subcellular resolution enables investigations of the heterogeneous distribution of saturated CE, which has been under-examined in previous studies. We also discovered that the birefringent crystals, previously found to be associated with NASH development, are predominantly composed of saturated CE.

**Conclusions:**

Our method allows for a detailed characterization of lipid composition in human liver tissues and enables further investigation into the potential mechanism of NASH progression.

## 1 Introduction

The molecular mechanisms driving the development of advanced fibrosing nonalcoholic steatohepatitis (NASH) and cirrhosis in a relatively small subset of persons with nonalcoholic fatty liver disease (NAFLD) are incompletely understood. Lipotoxicity is a prominent pathogenetic mechanism postulated to explain the progression of fatty liver disease from “simple steatosis” (or nonalcoholic fatty liver, NAFL) to NASH, accompanied by the development of hepatic necroinflammation and fibrosis. Lipotoxicity theories of NASH suggest that certain lipid molecules [e.g., cholesterol, ceramides, di-acylglycerol, or free fatty acids (FAs)] are responsible for initiating and/or driving the inflammation and fibrosis that characterizes NASH, whereas other lipid molecules [e.g., triglycerides (TAGs)] may be innocent bystanders that are present in NASH, even in relatively large quantities, but do not necessarily drive disease progression. The cellular (e.g., hepatocytes versus Kupffer cells versus stellate cells) and subcellular (e.g., within lipid droplets versus endoplasmic reticulum) localization of potentially harmful lipid species is also critical to understanding how lipotoxicity may contribute to NASH pathogenesis. We, and others, have been particularly interested in the potential role of cholesterol and cholesteryl esters (CEs) as critical lipotoxic molecules in NAFLD progression, including the implications of both their cellular and subcellular localization.^1^^–^^5^

Raman microscopy is a technique that has been applied to NAFLD only recently and has great potential in further clarifying the role of lipotoxicity in NASH because it can characterize both the type and spatial localization of relevant lipid molecules.^6^^–^^10^ Specifically, Minamikawa et al.^6^ applied Raman microscopy to visualize the distribution of lipid droplets in hepatocytes in NASH model mice liver tissues and demonstrated its capability of characterizing the molecular features of accumulated microvesicular and macrovesicular lipid droplets. Takemura et al.^7^ utilized spontaneous Raman microscopy to evaluate steatosis and retinol content in rat liver tissues at the nascent state of NAFLD. Diagnosing states of NAFLD in mice models can also be achieved by Raman micro-spectroscopy combined with machine learning algorithms.^8^^–^^10^ Our group has been involved in the development of hyperspectral stimulated Raman scattering (hsSRS) microscopy as an advancement over traditional Raman microscopy, which offers several advantages such as increased imaging sensitivity and acquisition speed.^11^^–^^14^ Moreover, the SRS signal has a linear dependence on the analyte concentration, making it an ideal tool for quantitative chemical composition analysis.^15^ Previous research has demonstrated the utility of SRS microscopy in investigating subcellular distributions and metabolic dynamics of lipid molecules in cells and tissues.^16^^–^^23^ By leveraging the spectral differences between lipid species, SRS microscopy has shown its potential in classifying different types of lipid molecules. Specifically, our lab has applied hyperspectral SRS imaging, i.e., acquiring SRS images at a series of vibrational frequencies, in the carbon–hydrogen (C–H) stretching region, between 2825 and $3050\;{\mathrm{cm}}^{-1}$, to quantitatively differentiate and localize CEs and TAGs in cells and mouse liver and adrenal gland tissues.^18^ Wang et al.^17^ demonstrated that fingerprint region (within 400 to $1800\;{\mathrm{cm}}^{-1}$) SRS imaging enables the classification of crystalline cholesterol, CEs, and TAGs in atherosclerotic arterial tissues. Moreover, SRS microscopy can also distinguish saturated lipid molecules (i.e., FA chains have all single carbon–carbon bonds) from unsaturated lipids (FA chains contain carbon–carbon double bonds) and determine the saturation level of specific lipid species. For instance, Jia et al.^23^ employed SRS imaging for quantifying TAGs of different saturation degrees based on the ratio between the =C–H band at $3010\;{\mathrm{cm}}^{-1}$ and the ${\mathrm{CH}}_{2}$ band at $2850\;{\mathrm{cm}}^{-1}$ in fibrotic liver tissues.

In this work, we apply a dual-band SRS imaging system that simultaneously acquires hyperspectral data from both the C–H stretching and fingerprint regions to accurately classify free cholesterol, saturated cholesterol ester, unsaturated cholesterol ester, and TAG. We also report our image analysis pipeline that enables characterizing the content and cellular localization of these lipid species in liver tissue sections from NAFL and NASH patients and compare them with traditional polarization microscopy used for identifying cholesterol crystals. We anticipate that the SRS imaging and analysis pipeline that we propose can be used in future studies to provide insights into how these lipid molecules might exert lipotoxic effects and contribute to the progression from NAFL to NASH.

## 2 Materials and Methods

### 2.1 Patients with Biopsy-Proven NAFL and NASH

Patient data, serum specimens, and liver tissue were derived from a biorepository at Veterans Affairs Puget Sound Healthcare System (VAPSHCS). This biorepository prospectively recruited patients undergoing clinically indicated liver biopsies and stored liver tissue that was flash-frozen in liquid nitrogen immediately after the liver biopsy. We identified patients with NAFLD based on histological hepatic steatosis on liver biopsy in the absence of hepatitis C virus (negative serum HCV antibody and HCV RNA), hepatitis B virus (negative serum HBV surface antigen), excessive alcohol consumption (dedicated alcohol questionnaire administered on the day of the liver biopsy), iron overload (hepatic stain and serum iron markers), or markers of autoimmune liver diseases. Liver slides were prospectively reviewed by a hepatopathologist who scored the grade of steatosis (1 to 3), inflammation (0 to 3), ballooning degeneration (0 to 2), and stage of fibrosis (0 to 4) according to the system proposed by Kleiner et al.^24^ We randomly selected frozen liver tissue specimens from patients with NAFLD who either had histological “isolated steatosis (NAFL)” or “fibrosing-NASH” defined as follows:

1. “Isolated steatosis”, also known as NAFL^25^ (n=5): defined by histological steatosis grades 1 to 3, inflammation grades 0 to 1, fibrosis stage 0, and ballooning degeneration grade 0.
2. “Fibrosing-NASH” (n=7): defined by histological steatosis grades 1 to 3, inflammation grades 1 to 3, fibrosis stages 1 to 3, and ballooning degeneration grades 1 to 2. We purposefully selected patients with NASH who had fibrosis because fibrosis is the histological feature most strongly associated with adverse long-term outcomes in patients with NAFLD.^26^^,^^27^

### 2.2 NAFLD Tissue Sectioning and Slide Preparation

A 2 to 3 mm piece of repository stored frozen human liver biopsies were snap-frozen in OCT compound. About 10  μm sections of the livers were cut on a cryostat, placed on glass slides, and stored at −70  deg. For polarization and SRS analysis, the slides were thawed at room temperature for 10 min and then coverslipped with Aquamount (ThermoFisher, Kalamazoo, Michigan, United States) using no. 1.5 cover glass. Sections were immediately viewed using a polarizing microscope followed by the SRS imaging system.

### 2.3 Dual-Band hsSRS Microscopy System

**Fig. 1:**
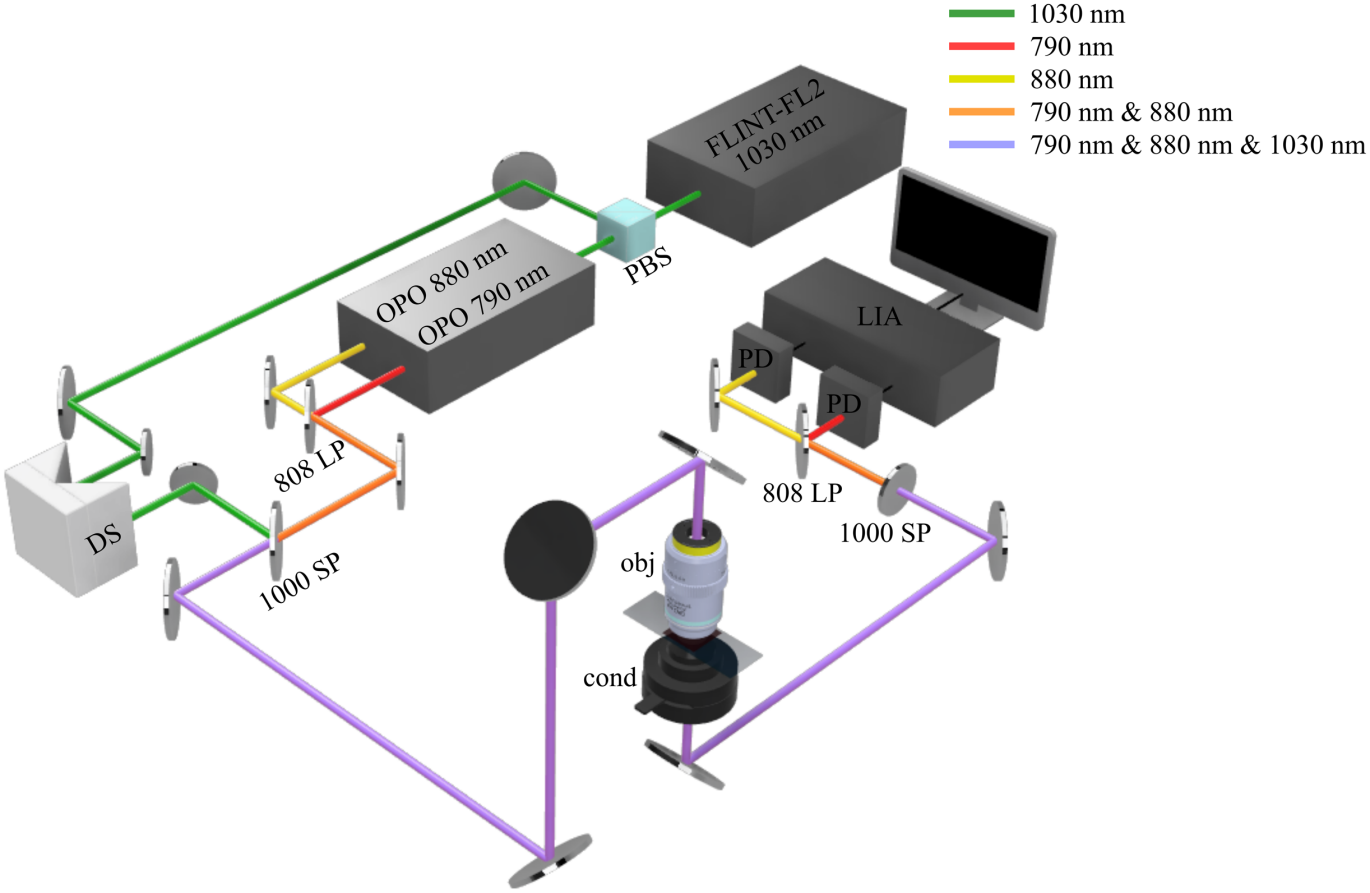
Schematic diagram of the dual-band hyperspectral SRS microscopy system for liver tissue imaging. The Stokes beam is generated directly by an ultrafast laser, FLINT-FL2, at a center wavelength of 1030 nm. The second harmonic of a 1030 nm pulse is used to pump two OPOs to generate two synchronized pump beams at 790 and 880 nm. The two pump beams are combined by an 808 nm long pass (808 LP) dichroic mirror. The three beams are then combined with a 1000 nm short pass (1000 SP) dichroic mirror and synchronized using a DS. The overlapped pulses are sent into a homebuilt upright microscope system (obj, objective; cond, condenser). The SRS signals are detected by two PDs and demodulated with a multichannel LIA.

The schematic diagram of the SRS imaging setup used for this project is shown in Fig. 1. We describe the dual-band hyperspectral SRS imaging method in detail in a prior work.^28^ The Stokes beam was generated by an ultrafast oscillator (Light Conversion FLINT-FL2) at a center wavelength of 1030 nm. The pump beams were generated using two home-built optical parametric oscillators (OPOs) at center wavelengths of 790 and 880 nm that probe the C–H and fingerprint regions, respectively. The synchronized femtosecond laser pulses were chirped to ∼2  ps using SF11 glass rods. The temporal delay between the pump and Stokes pulses was controlled by a motorized delay stage (DS; Zaber X-DMQ-AE). The combined beams were sent into a home-built microscope equipped with a 40× water immersion objective (Nikon N40XLWD-NIR, NA = 1.15). A polarizing beam splitter was used to control the polarization state of the incident beams at the sample plane. The polarizing beam splitter was configured to transmit linearly polarized light along the y-direction. At the sample, the power of both the pump and Stokes beams was 40 mW. The two pump beams were separated by a dichroic after the sample and detected separately using an amplified Si photodiode (PD), and the SRS signals were extracted by a dual-channel lock-in amplifier (LIA; Liquid Instruments Moku:Pro). All images collected were 512×512  pixels with a spatial resolution of ∼400  nm.

### 2.4 Spectral Unmixing and Lipid Classification

To determine the cellular distribution of free cholesterol, CE, and TAG, we collected the calibration standard SRS spectra of free cholesterol (Sigma-Aldrich), saturated CE [cholesteryl palmitate (13.1% CE, Sigma-Aldrich) and cholesteryl stearate (1.94% CE, Sigma-Aldrich)], unsaturated CE [cholesteryl linoleate (47.1% CE, Sigma-Aldrich) and cholesteryl oleate (20.3% CE, Sigma-Aldrich)], and TAG [1,3-dioleoyl-2-palmitoyl glycerol (11.7% TAG, Cayman Chemical), glyceryl trioleate (3.25% TAG, Sigma-Aldrich), 1-myristoyl-2-oleoyl-3-palmitoyl-rac-glycerol (0.911% TAG, Cayman Chemical), and 1,2,3-tri-heptadecenoyl glycerol (∼0.1% TAG, Cayman Chemical)]. We selected these CE and TAG calibration samples based on their high abundance (percentages shown in parentheses) in three human NAFLD tissue samples (two diagnosed as NAFL and one as NASH) measured with mass spectrometry in our laboratory.

We examined previously frozen liver tissue samples from 12 NAFLD patients, including 5 classified as NAFL and 7 as fibrosing-NASH. For each liver tissue slice, we acquired hyperspectral stacks of SRS images in both the C–H stretching region in the high wavenumber window, between 2800 and $3060\;{\mathrm{cm}}^{-1}$, and the C=C/C=O stretching region in the fingerprint window, between 1645 and $1780\;{\mathrm{cm}}^{-1}$. The lipid droplets were segmented from the surrounding non-lipid components, such as collagen, by thresholding the $2850/2930\;{\mathrm{cm}}^{-1}$ ratiometric image. A nonnegative linear least-squares spectral unmixing algorithm (MATLAB Isqnonneg) was applied to both the C–H and the fingerprint region spectra for spectral unmixing of each pixel in lipid droplets. The spectral unmixing results from the two spectral bands were combined to generate lipid composition maps.

### 2.5 Brightfield and Polarized Light Microscopy System

Liver tissue section slides were viewed by the Nikon Ni-E microscope using brightfield and polarization filters prior to hyperspectral SRS imaging. Images were taken using a Nikon color camera.

### 2.6 Assessment of Hepatic Free Cholesterol

Free cholesterol was extracted with water, methanol, and chloroform solvents (1:1:1 v/v/v). The chloroform fractions were dried using nitrogen gas, and the residues were dissolved in deuterated chloroform. NMR spectra were obtained using an 800 MHz Bruker Avance III spectrometer. Free cholesterol signal was identified, and its concentration was obtained using the residual solvent signal from the solvent as the internal reference.

### 2.7 Assessment of Other Hepatic Lipids

Lipids were extracted using dichloromethane/methanol after the addition of 54 isotope-labeled internal standards. The extracts were concentrated under nitrogen and reconstituted in 10 mM ammonium acetate in dichloromethane:methanol (50:50). Lipids were analyzed using the Sciex Lipidyzer platform consisting of a Shimadzu LC and AB Sciex QTRAP 5500 MS/MS system equipped with SelexION for differential mobility spectrometry (DMS). Multiple reaction monitoring was used to target and quantify lipids in positive and negative ionization modes with and without DMS.

## 3 Results

### 3.1 Quantitative Classification of Lipid Molecules

**Fig. 2:**
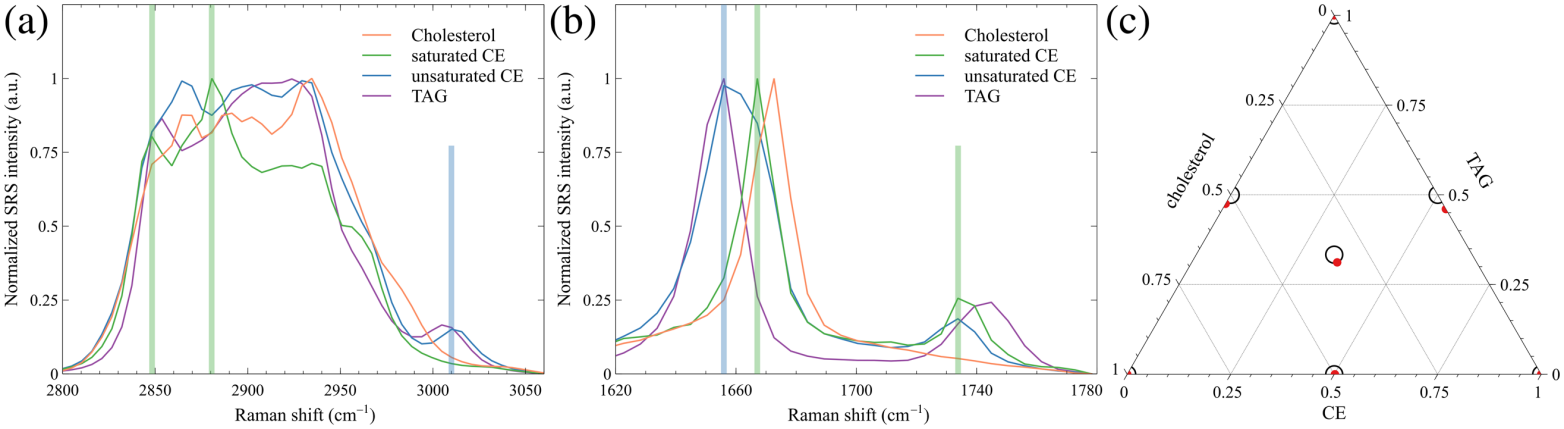
SRS spectra of cholesterol crystal, saturated CE, unsaturated CE, and TAG in the C–H region (a) and fingerprint region (b). Shaded lines: main spectral differences between saturated CE (green) and unsaturated CE (blue). (c) Ternary plot of the calculated fractions of three-component mixtures using SRS imaging and the spectral unmixing algorithm. Black circles: lipid standard mixture fractions. Red dots: calculated lipid fractions.

We obtained SRS spectra of several lipid standard samples as calibration standards to facilitate the identification of different lipid molecules. The normalized C–H region and fingerprint region SRS spectra of free cholesterol, CE, and TAG are demonstrated in Figs. 2(a) and 2(b), respectively. The SRS spectra of all imaged lipid standard samples are presented in Fig. S1 in the Supplementary Material. We observed clear spectral differences between free cholesterol, CE, and TAG. The relative peak heights in the 2850 to $2950\;{\mathrm{cm}}^{-1}$ region and around $3010\;{\mathrm{cm}}^{-1}$ are distinctive between different molecules. The spectral separations in the fingerprint are also distinct. Free cholesterol has a single sharp peak, resulting from C=C stretching, at $1673\;{\mathrm{cm}}^{-1}$ and no peak near $1740\;{\mathrm{cm}}^{-1}$, whereas both CE and TAG molecules contain C=O bonds that contribute to an additional peak at around $1740\;{\mathrm{cm}}^{-1}$. Compared with CE, the C=C Raman peak of TAG shifts lower, whereas the C=O shifts slightly higher. More interestingly, we observed a significant spectral difference between saturated CE and unsaturated CE. As indicated with shaded lines in Fig. 2, in the C–H region, saturated CE has a strong peak at around $2880\;{\mathrm{cm}}^{-1}$, a small peak at $2850\;{\mathrm{cm}}^{-1}$, and no peak at $3010\;{\mathrm{cm}}^{-1}$, whereas unsaturated CE has four peaks at 2865, 2900, 2930, and $3010\;{\mathrm{cm}}^{-1}$. Additionally, the saturated CE has a strong Raman peak at $1667\;{\mathrm{cm}}^{-1}$ in the fingerprint region (green shaded line), and the unsaturated CE has a peak near $1656\;{\mathrm{cm}}^{-1}$ (blue shaded line). These differences allow for spectral separation and identification of the saturated and unsaturated CE.

We then evaluated the accuracy of the classification and quantification of different types of lipid molecules with a ternary solution system. We performed SRS imaging and the linear least-squares spectral unmixing analysis on mixtures of cholesterol, cholesteryl oleate (representing CE), and glyceryl trioleate (representing TAG) at different ratios in ${\mathrm{CDCl}}_{3}$ solutions. The SRS spectra of cholesterol, unsaturated CE, and TAG in both the C–H stretching region (2800 to $3060\;{\mathrm{cm}}^{-1}$) and the fingerprint region (1645 to $1780\;{\mathrm{cm}}^{-1}$) were used for the unmixing analysis. The quantitative determination of lipid concentrations in three-component mixtures is shown in Fig. 2(c), with black circles representing the lipid standard mixture percentages and red dots indicating the calculated lipid percentages. The accuracies of the calculated concentrations are above 92%, with an average of 95.3%, validating that our imaging and analysis system provides a quantitative identification and classification of different lipid molecules based on their spectral information.

### 3.2 Lipid Composition and Cellular Localization in Human NAFLD Tissues

**Fig. 3:**
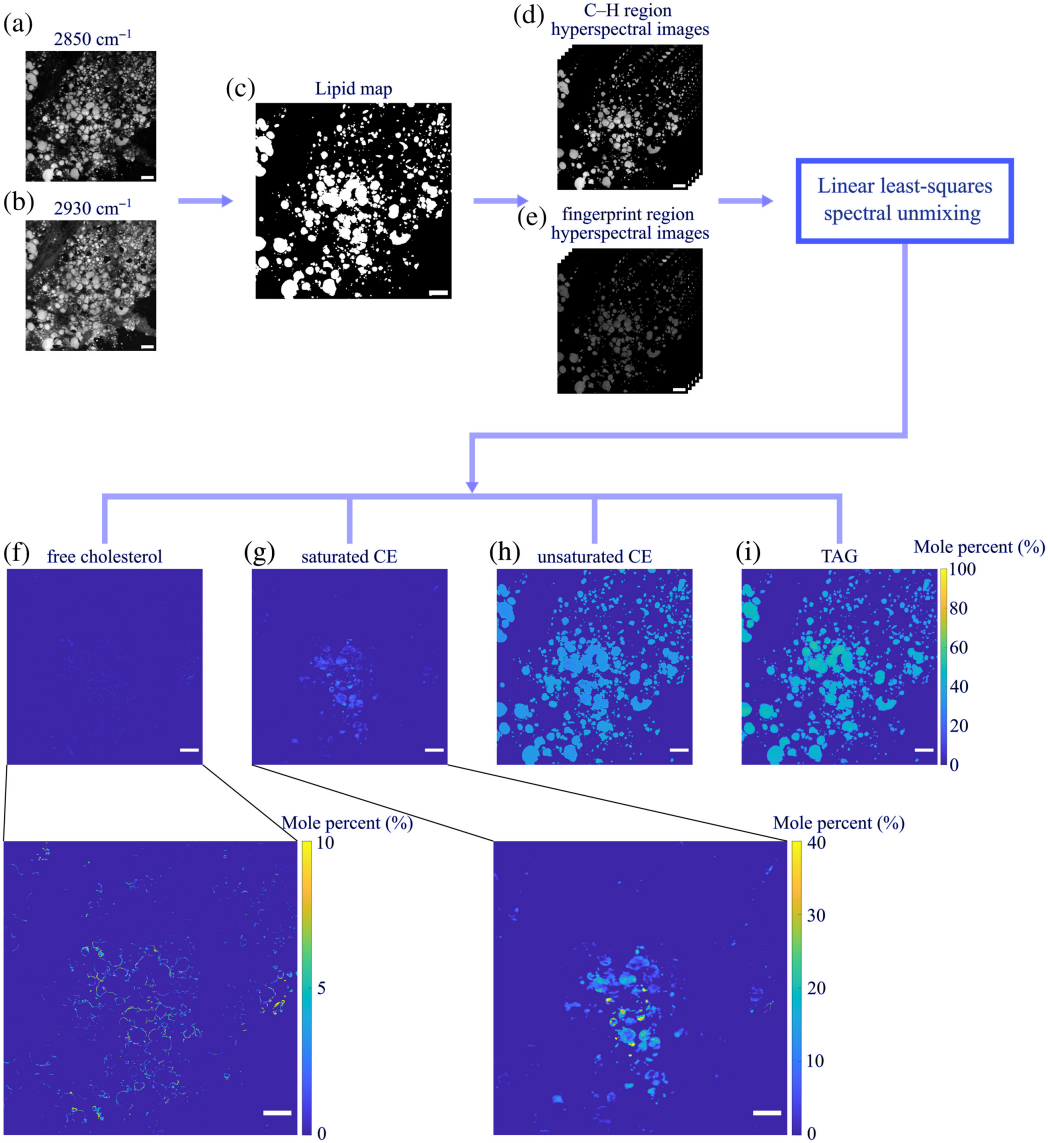
Schematic diagram of the image analysis pipeline and representative SRS images and lipid composition maps of a liver tissue section from a patient with NASH. From the SRS images at 2850 (a) and $2930\;{\mathrm{cm}}^{-1}$ (b), we use the $R_{2850/2930}$ ratiometric image to create a binary lipid map (c). We then perform the linear least-squares spectral unmixing analysis on both C–H and fingerprint region hyperspectral SRS images of lipid molecules (d) and (e) to generate mole percentage maps of free cholesterol, saturated CE, unsaturated CE, and TAG (f)–(i). The percentage maps of free cholesterol and saturated CE are also shown with a scale extending only from 0% to 10% (for cholesterol) and 0% to 40% (for saturated CE) rather than for 0% to 100% to make more apparent the distribution of these lipids that are present in much lower relative percentages than unsaturated CEs and TAGs. Scale bar: 50  μm.

We examined frozen liver sections from 12 NAFLD patients (7 NASH and 5 NAFL) via hyperspectral SRS imaging in the C–H and fingerprint regions, followed by linear least-squares spectral unmixing analysis. Our image processing pipeline is shown in Fig. 3. In the acquired SRS images, many non-lipid tissue components, predominantly proteins, contain abundant ${\mathrm{CH}}_{3}$ bonds and therefore generate strong signals at the ${\mathrm{CH}}_{3}$ peak at $2930\;{\mathrm{cm}}^{-1}$. By contrast, all lipid species are ${\mathrm{CH}}_{2}$ bond-rich and hence produce strong signals at the ${\mathrm{CH}}_{2}$ peak around $2850\;{\mathrm{cm}}^{-1}$.^29^ Leveraging this spectral difference, we can separate lipid molecules from their surrounding components by taking the ratio between SRS images at 2850 and $2930\;{\mathrm{cm}}^{-1}$, which highlights lipid contents and suppresses signals from other species. We generated a binary lipid map for each tissue by thresholding the $R_{2850/2930}$ ratiometric image [Fig. 3(c)]. After isolating lipid droplets from non-lipid backgrounds, including collagen and cell cytoplasm, we apply the linear least-squares spectral unmixing algorithm on each pixel of the lipid droplets in hyperspectral SRS images of both spectral regions to obtain the molar percentages of free cholesterol, saturated CE, unsaturated CE, and total TAG. We note that 100% is defined as the total concentration of these four aforementioned lipid species. We then plotted the mole percent measurements as percentage maps for each classified lipid type, as shown in Figs. 3(f)–3(i). To evaluate the performance of the fitting algorithm, we demonstrated three example pixel SRS spectra and their spectral unmixing algorithm fitted results in Figs. S2(a)–S2(c) in the Supplementary Material. The spectral unmixing results were determined as the sum of each individual lipid spectrum multiplied by its calculated percentage. The three pixels were selected from regions that were classified with higher percentages of TAG [Fig. S2(a) in the Supplementary Material], saturated CE [Fig. S2(b) in the Supplementary Material], or free cholesterol [Fig. S2(c) in the Supplementary Material]. Overall, the spectral unmixing results overlap well with the SRS spectra. We also generated fitting error maps for spectra in both the C–H and fingerprint regions individually [Fig. S2(d) in the Supplementary Material]. We evaluated the fitting error by the squared value of the fitting residual norm. A smaller residual norm value represents a more accurate fitting result. We also explored the distributions of TAG at different unsaturation levels by including spectra of TAG containing both saturated and unsaturated FA chains and TAG containing exclusively unsaturated FAs in the spectral unmixing algorithm. We demonstrated the comparison between lipid percentage maps generated by the two versions of the spectral unmixing algorithm in Fig. S3 in the Supplementary Material. Both spectral unmixing algorithm versions generated similar free cholesterol, saturated CE, and unsaturated CE percentage maps with comparable fitting error maps. The total TAG map generated in version 1 matches the sum of the two different unsaturation level TAG maps in version 2.

We discovered that, in the examined tissues, the localization of TAG and unsaturated CE is overall uniform across all lipid droplets. TAG is the dominant lipid species in the majority of lipid droplets, with an average percentage above 50% to 60%. We observed that distributions of TAG of different saturation levels are also uniform, with a slightly higher percentage of higher unsaturation degree TAG (Fig. S3 in the Supplementary Material). Interestingly, saturated CE and free cholesterol have much lower percentages and less uniform distributions compared with TAG and unsaturated CE in all tissues. We adjusted the contrast of both percentage maps from 0% to 100% to 0% to 40% and 0% to 10%, respectively, to better visualize saturated CE and free cholesterol in the representative field-of-view shown in Fig. 3. In Fig. 3(g), saturated CE forms a cluster below the center of the image but is not present in other lipid droplets. In Fig. 3(f), we observed that the algorithm classifies the outer periphery of most lipid droplets as free cholesterol. This pattern agrees with the localization of free cholesterol determined by filipin staining (Fig. S4 in the Supplementary Material). However, it is important to note that, because free cholesterol abundance is quite low, this percentage map is potentially susceptible to fitting errors. As illustrated in Fig. S2(c) in the Supplementary Material, the fingerprint region spectral unmixing result of a pixel selected from a region classified with higher free cholesterol shows a lower fitting accuracy with its SRS spectrum compared with the other pixels. The SRS spectrum is nosier in the 1700 to $1780\;{\mathrm{cm}}^{-1}$ region due to the lower SRS intensity from C=O vibration, which could lead to higher spectral unmixing inaccuracy. Additionally, as shown in the corresponding C–H region spectra fitting error map in Fig. S2(d) in the Supplementary Material, the squared residual norm value is higher around the edges of all lipid droplets. Based on lipidomics analysis data shown in Table S1 in the Supplementary Material, the average percentage of free cholesterol in a NASH tissue sample is at the level of 0.001%. If its distribution is only at the surface of droplets, as filipin staining suggested,^30^^,^^31^ the local concentration could be much higher and reach the detection limit of SRS. Nonetheless, quantifying free cholesterol with current SRS sensitivity may suffer from a lower accuracy compared with the other lipid species.

**Fig. 4:**
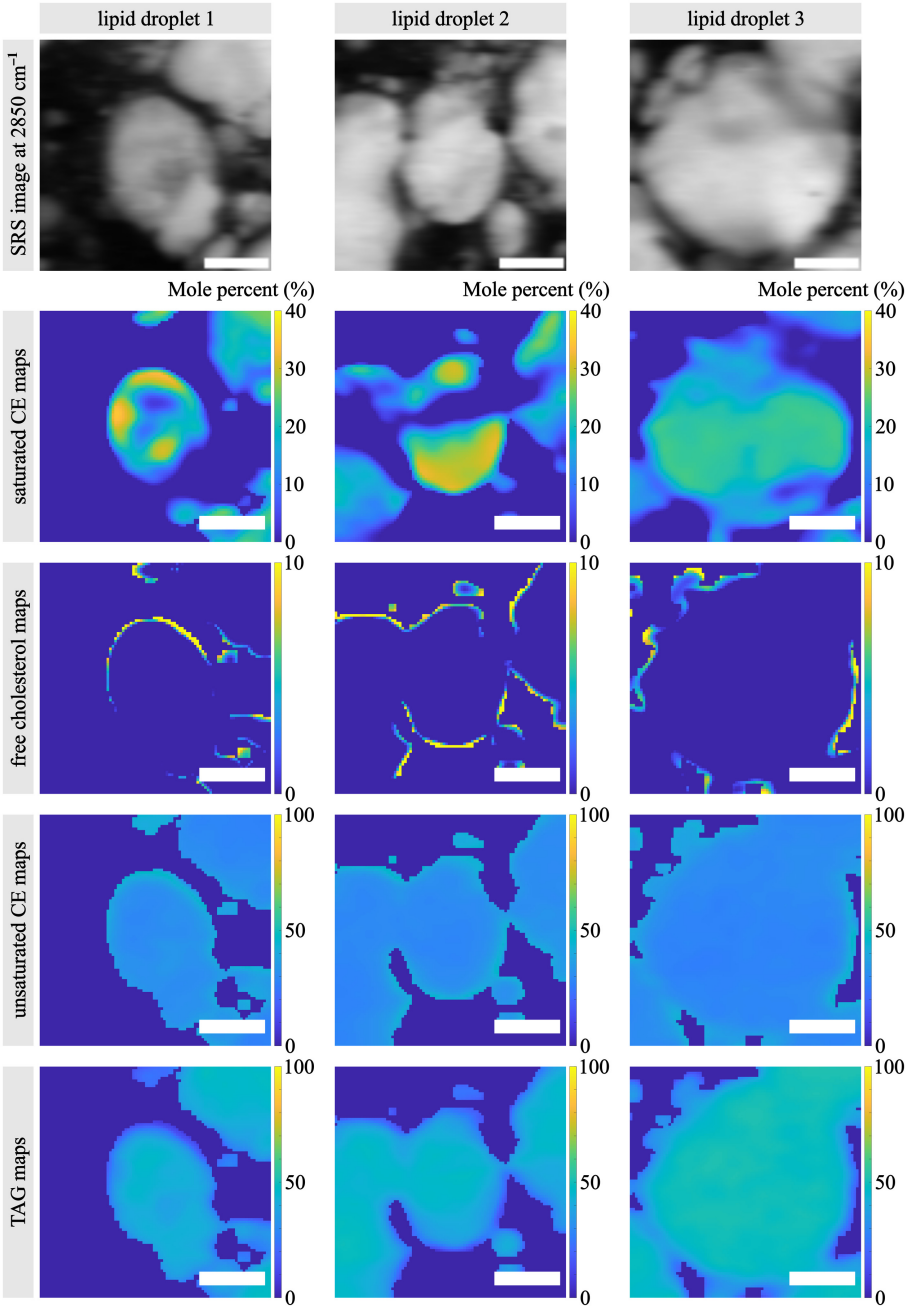
$2850\;{\mathrm{cm}}^{-1}$ SRS images and percentage maps of three representative lipid droplets selected from the frozen liver section of a patient with NASH. The three lipid droplets demonstrate homogeneous TAG and unsaturated CE compositions but heterogeneous free cholesterol and saturated CE compositions. Scale bar: 10  μm.

The submicron resolution of SRS microscopy allows us to investigate the subcellular distribution of analyzed lipid species in each lipid droplet. Three representative lipid droplets in an examined NASH tissue are shown in Fig. 4. Most interestingly, we discovered that saturated CE tends to self-associate into a heterogeneous subcellular localization pattern. Saturated CE molecules are observed to be predominantly near the boundary of the lipid droplet. In lipid droplets such as lipid droplet 1 in Fig. 4, saturated CE appears as arcs surrounding the central TAG and unsaturated CE core. In certain areas, as shown by lipid droplet 2, an enriched condensate of saturated CE can form crescent-like deposits. We hypothesize that these saturated CE molecules are inclined to self-aggregate to create a microenvironment in which they can easily interact with the lipid droplet envelope consisting of phospholipids and free cholesterol at the periphery. It is also possible that the crescent-like localization of saturated CE allows them to bind to abundant membrane proteins. Occasionally, further enrichment of saturated CE leads to a uniform deposit pattern across the lipid droplet, as shown in lipid droplet 3 in Fig. 4. Overall, the heterogeneity in the spatial distribution of specific lipid molecules confirmed that imaging techniques with a spatially resolved capability are essential for investigating the functions and interactions of various lipid species. We note that the polarization state of the incident laser beams at the sample plane may also contribute to the observed lipid distribution patterns. However, we found that the localization patterns of lipid molecules in general displayed random orientations and shapes across multiple samples, which suggests that they were not dominated by the laser polarization state.

### 3.3 Identifying the Origin of Birefringent Crystals in NAFLD Tissues

**Fig. 5:**
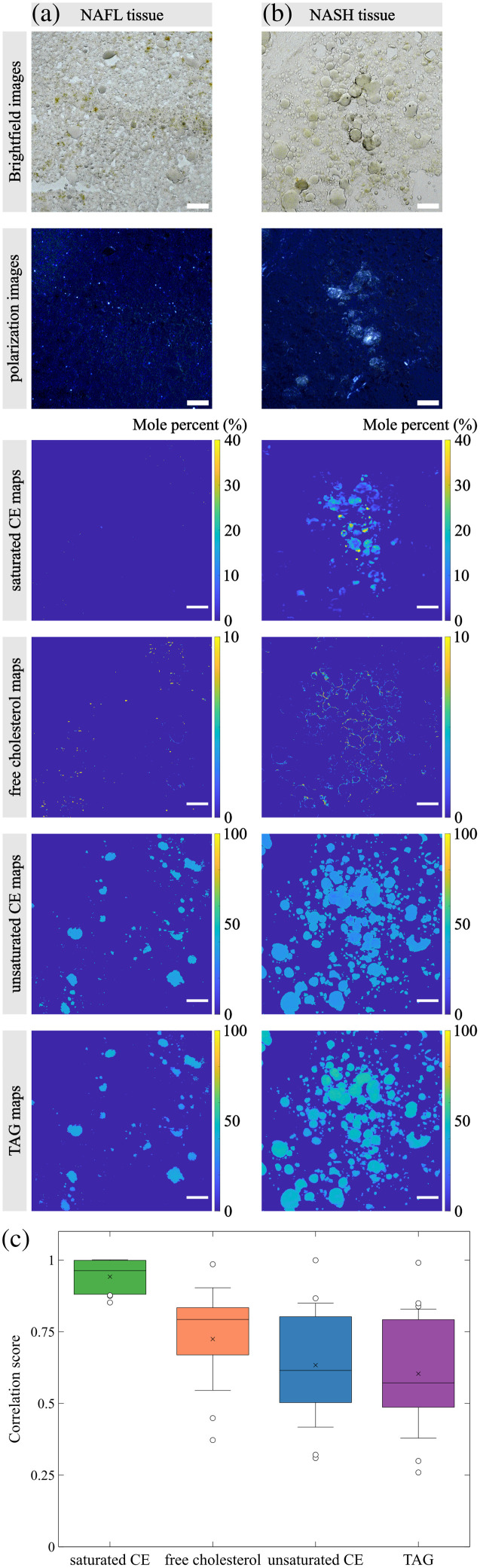
Brightfield, polarization images, and percentage maps of a NAFL tissue section region with low birefringent signals (a) and a NASH tissue section region with abundant birefringent crystals (b). Pixel percentage correlation plots between birefringent signals in polarization images and different lipid species. Scale bar: 50  μm. (c) Evaluation of spatial correlation between the polarization images and lipid percentage maps for 7 NAFLD tissues with standard deviations as error bars.

Polarized light microscopy has been used to investigate the association of cholesterol crystal formation with high cholesterol uptake in NAFLD tissues. It was hypothesized that cholesterol crystal leads to strong birefringence, which can be easily detected with polarized light microscopy. These crystals were indeed observed in both mouse tissue and human liver sections from patients with NASH.^4^^,^^32^ Furthermore, the birefringence signals of these crystals were found to be strongly correlated with NASH progression.^2^ However, the identity of those birefringent crystals was never conclusively determined due to a lack of spatially resolved chemical information. To investigate the chemical composition of the birefringent crystals, we performed SRS imaging and analysis on human tissue sections immediately after acquiring polarization images using polarized light microscopy. The brightfield, polarization images and SRS imaging generated percentage maps of the same regions for two NAFLD tissues are shown in Fig. 5. We selected a typical NAFL tissue section region with no birefringent signals (a) and a NASH tissue section region with abundant birefringent crystals (b). The NASH tissue contains a high accumulation of birefringent crystals in the center region and low birefringent signals in the surrounding lipid droplets. This pattern matches the saturated CE percentage map. The spatial difference between the polarization image and saturated CE percentage map is caused by the imaging tissue depth difference as the polarized light microscope has a much larger depth of focus compared with SRS microscopy. Similarly, the extremely low percentage of saturated CE in the selected NAFL tissue [Fig. 5(a)] agrees with its corresponding polarization image. The distributions of free cholesterol, unsaturated CE, and TAG suggested by their percentage maps have low spatial correlations with the strong birefringent signals. In both NAFL and NASH tissue images, the calculated free cholesterol accumulates around the periphery of lipid droplets, and the unsaturated CE and TAG localizations are uniform across the entire imaged regions.

We observed the same correlation trends in all imaged human liver tissue samples. We evaluated the spatial correlation between birefringent signals and different lipid molecules by converting the polarization images and lipid percentage maps to binary maps and comparing their pixel values. The correlation score was calculated for each tissue image as the number of matching pixels between the two binary images divided by the total number of pixels. A correlation score closer to 1 indicates a better matching between the two images. We report the correlation scores of the four lipid molecules for 7 NAFLD tissues in Fig. 5(c). The average correlation score of saturated CE is as high as 0.94, with a low standard deviation of 0.06, indicating a strong spatial correlation between birefringent signals in polarization images and saturated CE accumulation. The average correlation scores between birefringent signals and other lipid molecules, free cholesterol, unsaturated CE, and TAG, are 0.72, 0.63, and 0.60, respectively, with high standard deviations of 0.18, 0.22, and 0.22, respectively. These lower average correlation scores and larger variations indicate lower matchings between the distribution of birefringent signals and the localization of these lipid molecules. This reveals that these lipid species play less significant roles in birefringent crystal formation.

We plotted out the average percentages of different lipid species based on SRS-imaged regions of liver tissue sections from patients diagnosed with NAFL and NASH (Fig. S5 in the Supplementary Material). The two groups had no statistical significance for all lipid species, partially due to the limited sample size for each patient. More importantly, we observed abundant birefringent crystals in some regions of NAFL tissues and specifically selected these regions for SRS imaging, resulting in a selection bias with much higher percentages of saturated CE. These regions with strong birefringent crystals in NAFL may indicate the development of NASH. In homogeneous sample measurements, such as lipidomics analysis, these early signs of NASH development are susceptible to being overlooked because of their low percentages compared with the rest of the tissue. With a larger tissue size and unbiased sampling, our SRS method is suitable for detecting regions with abundant saturated CE to provide insights into the progression from NAFL to NASH.

## 4 Discussion

Understanding the molecular mechanisms underlying the disease progression from NAFL to NASH is crucial for both the prevention and the treatment of NASH. Lipotoxicity induced by different lipid molecules is thought to play a critical role in the development and progression of NASH. Lipidomics analysis is a well-established technique for quantifying lipid composition, but it does not provide spatial information and therefore hinders the investigation of lipid localization heterogeneity in the analyzed specimens. In comparison, SRS microscopy offers both spatial (cellular and subcellular) and chemical information on lipid species classification and quantification. In this work, we presented an SRS method that combines C–H and fingerprint regions to characterize the distribution of different lipid species in liver tissue sections from NAFLD patients. We note that the dual-band hyperspectral SRS system is not a fundamental requirement for the study as the same analysis pipeline can be applied to images acquired with conventional single-band SRS systems by the tuning laser wavelength. However, the simultaneous acquisition of SRS images in the two separate spectral regions without wavelength tuning improves the imaging speed two-fold and minimizes imaging inaccuracy caused by sample movement, focus drift, or laser power fluctuation. By collecting spatial and spectral information from hyperspectral SRS imaging in both the high wavenumber C–H stretching region and the fingerprint C=C/C=O stretching region, we demonstrated spatially resolved mapping of free cholesterol, saturated CE, unsaturated CE, and TAG distribution in lipid droplets. We developed an image acquisition and processing pipeline that allows us to explore the cellular and subcellular localization patterns of these different lipid molecules.

We found that TAG is the most abundant lipid species and distributes uniformly across all hepatocyte lipid droplets in the human NAFLD tissues, followed by unsaturated CE. Saturated CE has a much lower mole percentage than TAG and unsaturated CE. These results agree with lipidomics analysis. Importantly, we found that only saturated CE and free cholesterol have a heterogenous distribution pattern: saturated CE molecules tend to self-aggregate around the periphery of a small percentage of lipid droplets with further enrichment leading to a relatively uniform localization within the lipid droplets, whereas the outer periphery of lipid droplets is the most probable location for free cholesterol to accumulate. The cholesterol distribution is supported by filipin staining, but quantification is susceptible to fitting errors due to its low abundance and thus requires further validation.

We then used the developed pipeline to investigate the composition of birefringent liquid crystals that have been previously associated with NASH progression.^2^^,^^4^ We discovered a high spatial correlation between the birefringent crystals and saturated CE by comparing SRS images and polarization images of the same tissues. This finding is supported by previous studies reporting that CE molecules can form liquid crystalline lattices in lipid droplets.^33^ Indeed, we have observed the emergence of liquid crystals with typical “Maltese crosses” in liver biopsies of animal models of NASH preceding the onset of inflammation and fibrosis.^32^ The strong spatial correlation indicates that the main composition of these birefringent liquid crystals is saturated CE, suggesting that the presence and the fate of saturated CE (e.g., lipolysis, phase equilibrium, and phase transition of cholesterol) may also play a vital role in NASH progression. It is important to note that the spatial correlation between the polarization and SRS images is reduced by two factors: the imaging depth mismatch between the polarized light and SRS microscopy systems and other components, mainly collagen, also generating birefringent signals in the polarization images. In polarization images, the collagen signals can overlap with signals of the birefringent crystals of interest, hindering the visualization of crystal distribution. Our developed SRS imaging and analysis system addressed this problem by separating signals from non-lipid components, such as collagen, to only focus on lipid molecules. With the spatially resolved capability of SRS imaging, we observed that some small regions in clinically diagnosed NAFL tissue sections contain accumulated saturated CE, which are potential indicators of NASH development. Future studies will focus on elucidating the origin of saturated CE aggregation in lipid droplets and its role in the pathogenesis of NASH. The SRS microscopy platform can be used to test specific hypotheses of lipotoxicity and may have potential clinical applications in the future if SRS “markers” associated with clinical outcomes are identified.^8^^–^^10^

Our work has some limitations. We only focused on free cholesterol, cholesterol esters (saturated and unsaturated), and TAGs (of different saturation levels) in this work. Due to their lower percentages in examined NAFLD tissues (∼3% TAG), the classification of TAG containing only saturated FAs, i.e., saturated TAG, was not included in this work. The Raman spectral difference between saturated TAG and other lipid species has been explored in previous studies. As Czamara et al.^34^ reported, although saturated TAG has a similar Raman spectrum as saturated CE in the C–H stretching region with subtle peak shifts and relative peak intensity differences, they have significant spectral differences in the C=C/C=O stretching region in the fingerprint window. Specifically, saturated TAG has no peak near $1660\;{\mathrm{cm}}^{-1}$ due to a lack of C=C bond and has double peaks at 1743 and $1729\;{\mathrm{cm}}^{-1}$.^34^ Leveraging these spectral differences, the classification of saturated TAG can also be achieved using the SRS spectral unmixing algorithm. Moreover, future studies can expand the SRS technique to explore other lipid species, such as free FAs and phospholipids. However, it is important to note that there is a limitation in the number of lipid species that can be classified by SRS imaging depending on the spectral difference between different lipid molecules. Increasing the spectral window in the fingerprint region may help, but due to the limited sensitivity of SRS imaging, it is difficult to reliably quantify lipid species with a molar percentage at the level of or below 5%. Therefore, although SRS imaging is not a substitute for other techniques such as lipidomics in the comprehensive profiling of lipids, it can be used in conjunction with lipidomics to provide complementary spatial information and a more detailed characterization of the lipid composition in NAFLD tissues. In conclusion, we believe that the SRS imaging and analysis pipeline that we developed provides a valuable tool for characterizing the spatial distribution of different lipid species in human liver sections, which appear to be inhomogeneous in NAFL/NASH. It offers great potential for gaining new insights into the molecular mechanisms of the progression from NAFL to NASH.

## Data Availability

All data are available from the authors upon request.
