# Supplementary material for: Discrimination of lipid composition and cellular localization in human liver tissues by stimulated Raman scattering microscopy
Source: J Biomed Opt. 2024 Jan 24;29(1):016008. doi: 10.1117/1.JBO.29.1.016008 (PMC10807871; doi:10.1117/1.JBO.29.1.016008)
Supplement: Supplementary file 1 [file JBO_029_016008_SD001.pdf]

Supplementary Material

Discrimination of lipid composition and cellular localization in human liver tissues by stimulated Raman scattering microscopy

Fiona Xi Xu[a], George N. Ioannou*[b,c,d], Sum P. Lee[c], Christopher Savard[b,c,d], Christian L. Horn[e], and Dan Fu*[a]

[a] Department of Chemistry, University of Washington, Seattle, WA 98195, USA
[b] Division of Gastroenterology, Department of Medicine, Veterans Affairs Puget Sound Health Care System, Seattle, WA, USA
[c] Division of Gastroenterology, Department of Medicine, University of Washington, Seattle, WA, USA
[d] Research and Development, Veterans Affairs Puget Sound Health Care System, Seattle, WA, USA
[e] Division of Gastroenterology and Hepatology, Department of Medicine, San Antonio Military Medical Center, Fort Sam Houston, TX, USA

*** Corresponding Authors:** danfu@uw.edu, george.ioannou@va.gov

Table of contents

- **Supplemental Figure S1**: SRS spectra of cholesteryl esters and triglycerides of different saturation levels.
- **Supplemental Figure S2**: Pixel spectral unmixing algorithm fitting examples and error maps.
- **Supplemental Figure S3:** Comparison of two spectral unmixing algorithm versions.
- **Supplemental Figure S4**: Filipin staining for free cholesterols in liver tissue.
- **Supplemental Figure S5:** Comparison of lipid species percentages in NAFL versus NASH liver tissue sections.
- **Supplemental Table S1**: Average NAFL and NASH human liver biopsy hepatic lipid composition measured by lipidomics analysis.

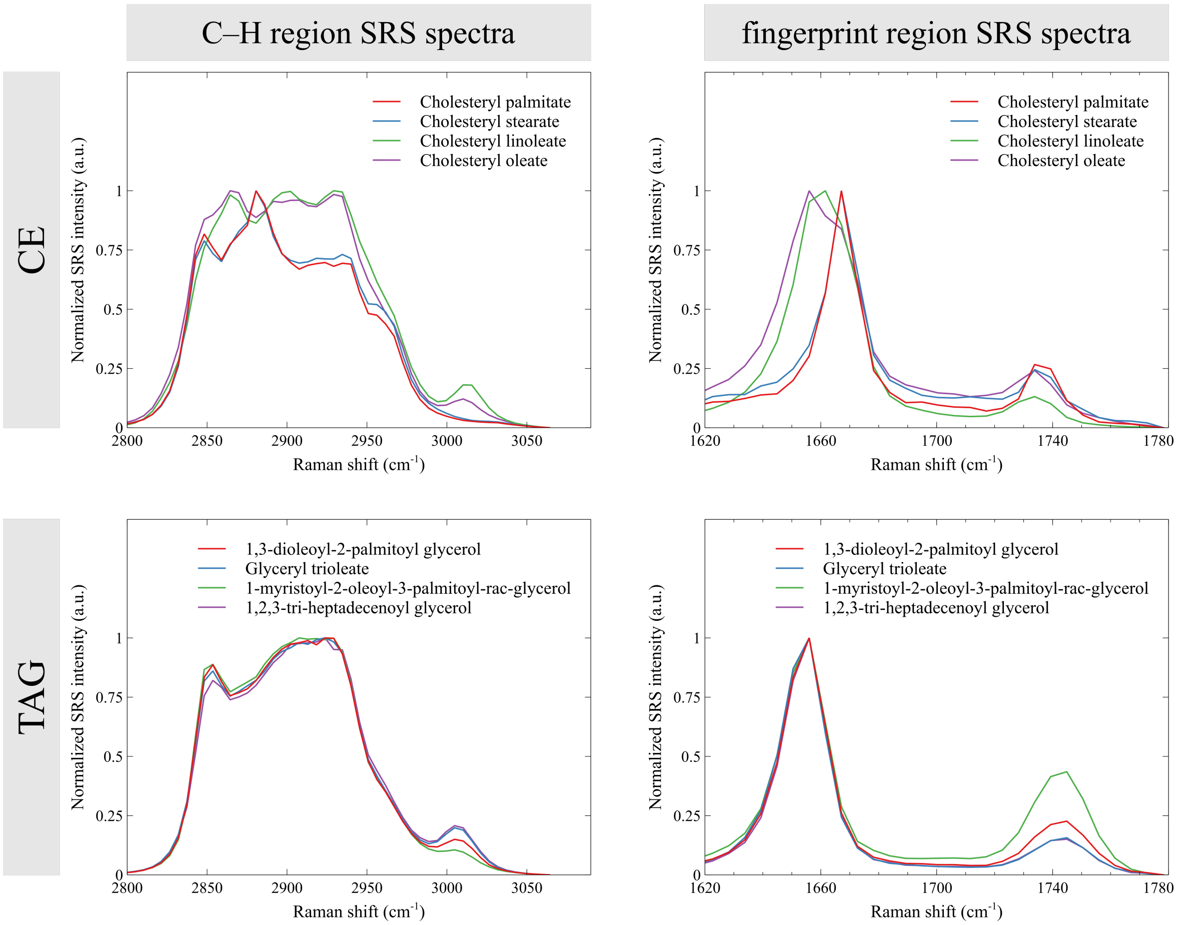

Supplemental Figure S1: *C–H region and fingerprint region SRS spectra of cholesteryl esters (CE) and triglycerides (TAG) of different saturation levels.*

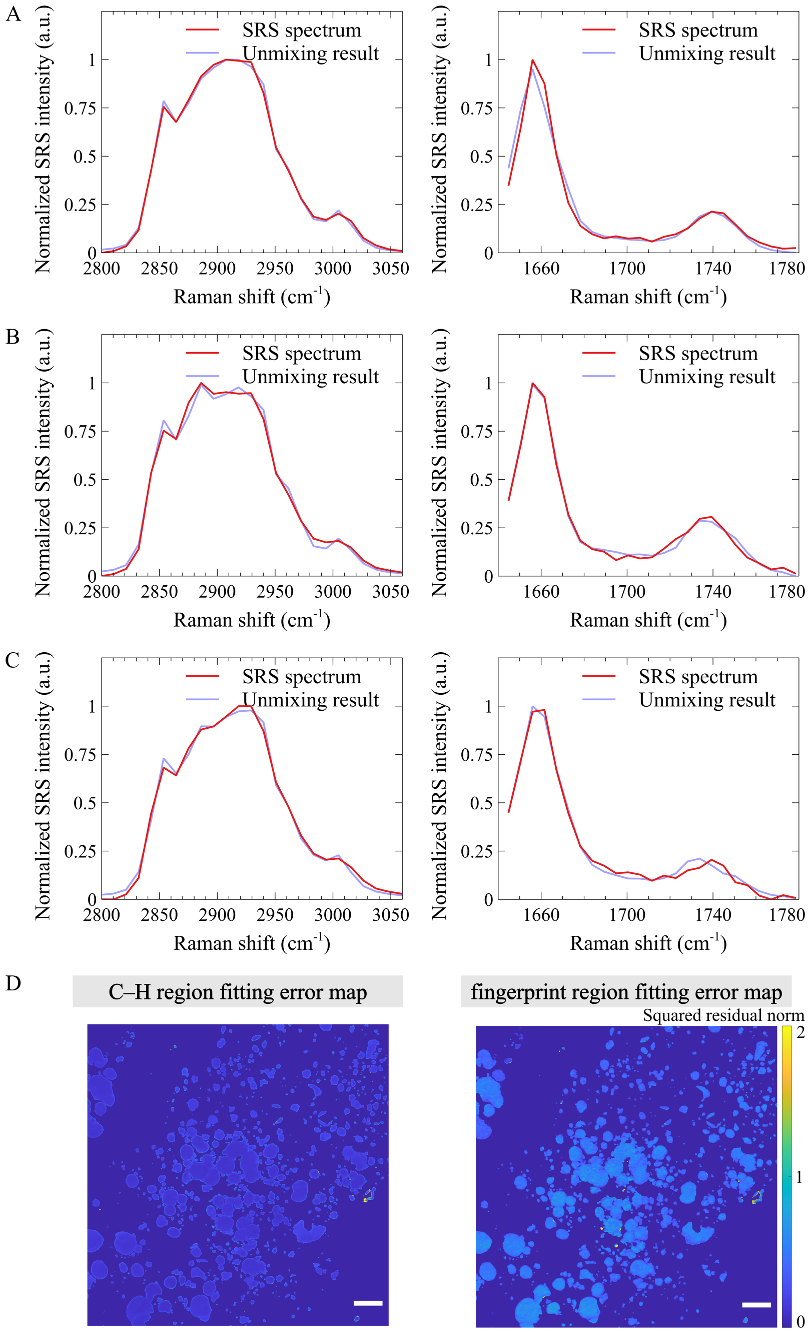

Supplemental Figure S2: *Representative pixel SRS spectra and corresponding spectral unmixing algorithm fitting results (A-C) and fitting error maps (D) of a representative liver tissue section from a patient with NASH. The corresponding free cholesterol, saturated CE, unsaturated CE, and total TAG percentage maps are shown in* ***Figure 3F - I****. (A) SRS spectra of a pixel selected from TAG-dominated region. (B) SRS spectra of a pixel selected from saturated CE-dominated region. (C) SRS spectra of a pixel selected from high cholesterol percentage region. The intensity of each pixel in the fitting error maps (D) represents the square of fitting residual norm. A lower square residual norm value means more accurate fitting. Scale bar: 50 µm.*

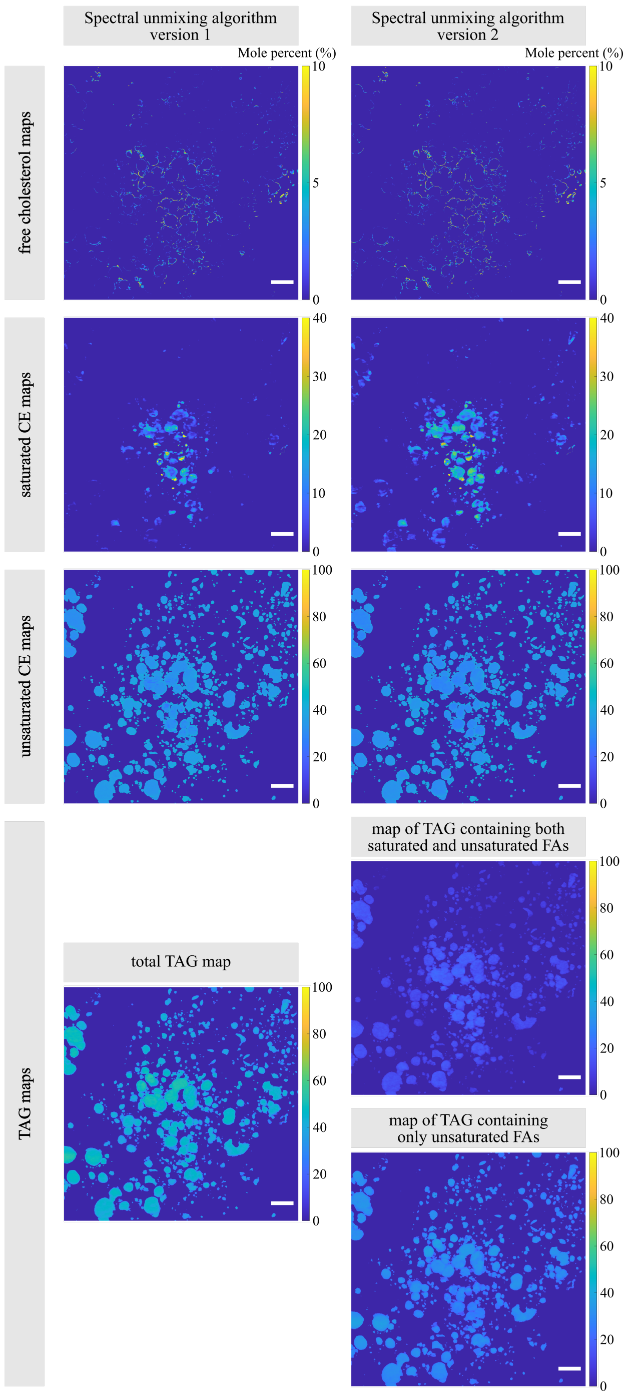

Supplemental Figure S3*: Comparison of two spectral unmixing algorithm versions with a representative liver tissue section from a patient with NASH. Spectral unmixing algorithm version 1: SRS spectra of free cholesterol, saturated CE, unsaturated CE, and average TAG (****Figure 2****) were used for spectral unmixing. Spectral unmixing algorithm version 2: spectra of free cholesterol, saturated CE, unsaturated CE, and two TAG of different unsaturation levels (****Supplemental Figure S1****, 1-Myristoyl-2-Oleoyl-3-Palmitoyl-rac-glycerol as TAG containing both saturated and unsaturated FAs, and 1,2,3-Tri-Heptadecenoyl Glycerol as TAG containing only unsaturated FAs) were used for spectral unmixing. Scale bar: 50 µm.*

Filipin staining for free cholesterol

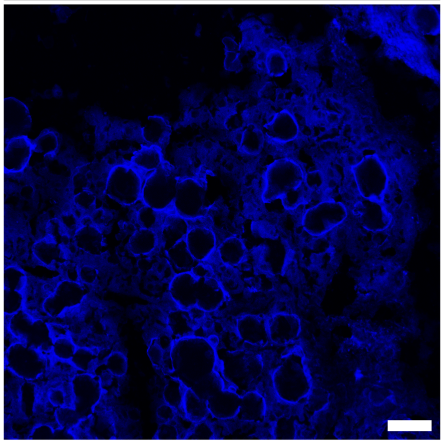

Supplemental Figure S4*: Filipin staining for free cholesterols in the same liver tissue (different section) as* ***Figure 3*** *from a patient with NASH. Free cholesterol tends to localize around the outer periphery of lipid droplets. Scale bar: 50 µm.*

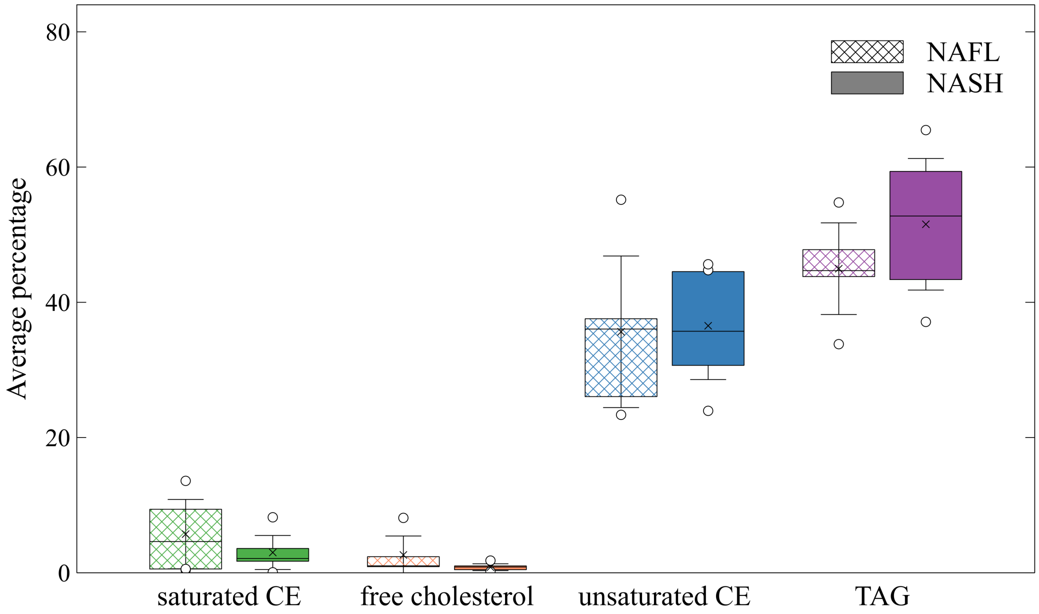

Supplemental Figure S5*: Average percentages of different lipid species in the imaged regions of liver tissue sections from patients diagnosed with NAFL versus NASH.*

Group	CE	Free Cholesterol	TAG	DAG	FFA
	nmol/ mg liver	nmol/ mg liver	nmol/ mg liver	nmol/ mg liver	nmol/ mg liver
**NAFL**					
Average	2065.303	1.297	54328.152	5679.604	22542.003
St Dev	1108.389	0.810	38711.715	3727.636	8573.912
% of total lipids	1.847	0.001	48.581	5.079	20.157
**NASH**					
Average	3040.530	1.179	92521.356	9542.998	31285.772
St Dev	1753.570	0.578	49763.588	5249.500	14950.443
% of total lipids	1.831	0.0007	55.708	5.746	18.838
**Ratio of NASH/NAFL**	1.472	0.909	1.703	1.680	1.388
t-test	0.104	0.663	0.035	0.039	0.084

***Supplemental Table S1**: Average NAFL and NASH human liver biopsy hepatic lipid composition measured by lipidomics analysis. Statistical analysis was performed using the Student t-test. CE: cholesteryl ester; TAG: triglyceride; DAG: diglyceride; FFA: free fatty acid.*
